# Cesarean section in China, Taiwan, and Hong Kong—A safe choice for women and clinicians?

**DOI:** 10.1371/journal.pmed.1002676

**Published:** 2018-10-16

**Authors:** Mairead Black, Sohinee Bhattacharya

**Affiliations:** Obstetrics and Gynaecology, Institute of Applied Health Sciences, University of Aberdeen, Aberdeen, United Kingdom

## Abstract

Mairead Black and Sohinee Bhattacharya discuss research findings on preferences for cesarean delivery in Asian settings and share their Perspective on facilitating woman-centered birth choices in China following the end of the one-child policy.

Recent high cesarean section (CS) rates around the world have sparked intense interest in the underlying drivers, partly to inform efforts to reduce CS rates. This week in *PLOS Medicine*, Long and colleagues report on these trends from multiple perspectives in mainland China, Taiwan, and Hong Kong, where recent social, political, and economic changes have impacted birth plans [1]. These settings—with respective CS rates of 34.9%, 27.4%, and 35%—reside near the top of the CS birth rate tables, alongside Brazil, Egypt, and Turkey, each reporting rates of over 45% [2].

Using meta-analysis and meta-ethnography to synthesise quantitative and qualitative studies respectively, Long and colleagues assessed the proportion of women who prefer CS at various time points in pregnancy and the factors influencing CS preferences among women, their family members, healthcare providers, and policy makers. The prospectively registered study stands out among related work because of the meticulous efforts made to understand and contextualise how nonclinical considerations lead to plans for CS births. After screening 22,932 citations identified in a systematic search, the authors included 66 papers, 45 of which were Chinese-language publications. In addition to detailing why many CS plans are made, the authors also report a recent shift towards placing greater value on vaginal birth in studies from mainland China. The latter observation supports previously reported impressions that removal of China’s one-child policy, in addition to multiple strategies to reduce CS rates, has led to an increase in vaginal births in women who previously gave birth by CS [3].

## Socioeconomic factors for women and clinicians

Maternal preference for CS assessed in early pregnancy played a minor part in explaining rising CS trends, as previously found in countries with high CS rates [4]. However, interactions with healthcare providers during pregnancy emerge as a contributor towards 1 in 5 women in the third trimester preferring a CS birth. A further proportion appears to be explained by decisions made by clinicians. In keeping with evidence from Turkey—where in 83.1% of cases women described a plan for CS as being their obstetrician’s decision—Long and colleagues identified that not only is CS perceived to reduce litigation risk but some clinicians in mainland China may be motivated to perform CS to increase financial revenue, even when this is in direct conflict with clinical guidance [5].

Economic growth and women’s empowerment, known drivers of CS births in resource-constrained settings, were important influences in mainland China, Hong Kong, and Taiwan [6]. In addition to low birth rates and rising maternal age, their growing economies mean many women are financially independent—the cost of a CS birth in Taiwan equates to the average monthly income—making CS easily affordable [7]. These observations are relevant to other settings in which similar socioeconomic forces are at play and likely explain why introducing equal financial reward for CS and vaginal birth led to a lowered elective CS rate in Taiwan but no change in women’s demand for CS [7].

## CS as the ‘safe’ option and conducive to woman-centred care

Long and colleagues found that belief in the ‘safety’ of CS birth underpinned women’s CS preferences in early pregnancy, not least when women felt they ‘had one chance to get it right’ under China’s one-child policy. Women–clinician relationships were critical to evolving CS choices during pregnancy, in which loss of faith in a provider due to a perceived lack of woman-centred care led to CS requests in a bid to avoid negative intrapartum experiences.

Multiple women described CS as the ‘safe’ choice for birth; quotes detailed CS as a means to avoid ‘any risk’ to their baby, whether ‘immediate complications’ or ‘longer-term child-development’ issues. Such perceptions of CS as being a safer birth option than aiming for vaginal birth are somewhat at odds with the 2015 World Health Organization’s statement on CS, which cites maternal and offspring ‘risk’ associated with nonmedically indicated CS [8]. However, the practice of attributing greater risk to a single CS than to a single attempt to achieve vaginal birth has been challenged by research arising from mainland China. Utilising the combination of large population databases of birth-related events and a high CS-on-maternal-request (CDMR) rate, researchers reported outcomes of over 66,000 first births in Shanghai between 2007 and 2013 [9]. Reduced offspring birth trauma, neonatal infection, meconium aspiration syndrome, and hypoxic ischaemic encephalopathy followed CDMR, with no difference in risk of serious maternal complications when compared with a plan for vaginal birth. Offspring born by CDMR were more likely to experience respiratory-distress syndrome, in keeping with existing evidence on elective CS [10]. Overall, these findings suggests that, where women are certain of their plan to have only one child, those with similar characteristics in equivalent healthcare settings may be justified in choosing CS on safety grounds. Going forward, however, families and clinicians in mainland China should be aware that with the recent removal of China’s one-child policy, apparently well-informed birth plans may become invalid when a previously unplanned future pregnancy arises. Adverse consequences of a previous CS such as abnormally adherent placenta and CS scar rupture are rare but can be devastating and so should be weighed up against potential benefits of the initial CS.

Long and colleagues aimed to identify mechanisms through which unnecessary CS may be reduced in mainland China, Hong Kong, and Taiwan, where ‘unnecessary’ is defined as ‘not clinically indicated’. However, as their study demonstrates, some women choose CS based on their own beliefs and value judgements, not the judgement of their clinician, such that the overall study aim risks undermining the importance of women’s preferences. Instead of strategies to lower CS rates per se, efforts should move towards ensuring that women who prefer vaginal birth feel safe to aim for this mode of delivery in collaboration with their providers and that financial incentives for clinicians are equal for CS and vaginal birth. The agenda to lower CS rates appears to be driven by WHO’s position statement, which cites a lack of evidence for reduction in maternal and infant mortality at the population level for CS rates above 10%–15% [8]. However, the WHO statement does not reflect the quality-of-life outcomes that appear to be important to women in mainland China, Taiwan, and Hong Kong such as birth experience and sexual function. Long and colleagues’ findings demonstrate that women and clinicians in these settings who plan CS may be voting with their feet to optimise both perceived safety and quality-of-life outcomes. In the United Kingdom and Singapore, where recent person-centred legal developments mean that informed consent to give birth requires that women are informed of (1) risks she considers to be important and (2) reasonable available options, decisions for CS based upon quality-of-life outcomes appear legitimate yet highlight the gulf between WHO priorities (saving lives) and those of women and clinicians making individual birth plans [11,12].

## China’s importance in future research

It is clear from the work of Long and others that China’s recent one-child policy has affected attitudes towards CS birth, and the end to the policy demands a fresh look at how future pregnancy and long-term outcomes of CS birth are studied and shared with women. Information about the cumulative and long-term risks and benefits of a planned CS across more than one pregnancy are sadly lacking. China is in a strong position to use its high CDMR rates and its population-based birth registries to support studies of birth outcomes beyond mortality and to engage with women to identify outcomes that are important to them. Such a truly woman-centred approach would facilitate birth choices being made in the full knowledge of the balance of risks and benefits.

## Abbreviations

CDMR: cesarean delivery on maternal request
CS: cesarean section

## References

[1] Long Q, Kingdon C, Yang F, Renecle MD, Jahanfar S, Bohren MA, et al Prevalence of and reasons for women's, family members' and health professionals' preferences for caesarean section in China: A mixed-methods systematic review. PLoS Med. 2018;15(10):e1002672 10.1371/journal.pmed.1002672 PMC6191094 30325928

[2] Betrán AP, Ye J, Moller A-B, Zhang J, Gülmezoglu AM, Torloni MR. The Increasing Trend in Caesarean Section Rates: Global, Regional and National Estimates: 1990–2014. PLoS ONE. 2016;11(2):e0148343 10.1371/journal.pone.0148343 26849801 PMC4743929

[3] Mazzoni A, Althabe F, Liu N, Bonotti A, Gibbons L, Sánchez A, et al Women’s preference for caesarean section: a systematic review and meta‐analysis of observational studies. BJOG 2011;118:391–399. 10.1111/j.1471-0528.2010.02793.x 21134103 PMC3312015

[4] Mu Y, Li X, Zhu J, Liu Z, Li M, Deng K, et al Prior caesarean section and likelihood of vaginal birth, 2012–2016, China. Bull World Health Organ. 2018;96:548–557. 10.2471/BLT.17.206433 30104795 PMC6083396

[5] Kisa S, Zeyneloğlu S. Opinions of women towards cesarean delivery and priority issues of care in the postpartum period. Appl Nurs Res. 2016 5;30:70–5. 10.1016/j.apnr.2015.11.004 27091257

[6] Khan MN, Islam MM, Rahman MM. Inequality in utilization of cesarean delivery in Bangladesh: a decomposition analysis using nationally representative data. Public health. 2018;157:111–20. 10.1016/j.puhe.2018.01.015 29518616

[7] Chen CS, Liu TC, Chen B, Lin CL. The failure of financial incentive? The seemingly inexorable rise of cesarean section. Soc Sci Med. 2014 1;101:47–51. 10.1016/j.socscimed.2013.11.010 24560223

[8] World Health Organization. WHO statement on caesarean section rates. 2015 [cited 2018 Aug 7]. World Health Organization, Geneva. Available at: http://www.who.int/reproductivehealth/publications/maternal_perinatal_health/cs-statement/en/

[9] Liu X, Landon MB, Cheng W, Chen Y. Cesarean delivery on maternal request in China: what are the risks and benefits? Am J Obstet Gynecol 2015;212:817.e1–9. 10.1016/j.ajog.2015.01.043 25640048

[10] Hansen A, Wisborg K, Uldbjerg N, Henriksen T. Risk of respiratory morbidity in term infants delivered by elective caesarean section: cohort study. BMJ 2008;336:85 https://www.bmj.com/content/336/7635/85.short 10.1136/bmj.39405.539282.BE 18077440 PMC2190264

[11] The UK Supreme Court. Judgement: Montgomery (appellant) vs Lanarkshire Health Board (respondent) (Scotland). 2015 [cited 2018 Aug 7]. Available at: https://www.supremecourt.uk/cases/docs/uksc-2013-0136-judgment.pdf

[12] Menon S, Chuan VT. Singapore modifies the UK Montgomery Test and changes the standard of care doctors owe to patients on medical advice. J Bioeth Inq. 2018 Jun;15(2):181–183. 10.1007/s11673-018-9868-3 29968015

